# The association between early-life nutrition and palatine tonsil grading in preschool children: a cross-sectional study

**DOI:** 10.3389/fnut.2026.1789543

**Published:** 2026-03-19

**Authors:** Guangwei Guo, Wanyun Yan, Ruifa Zhou, Lianhua Lü, Yitong Lu, Yuyong Pan, Jianhui Luo, Chengquan Wu, Meilian Chen, Dongmin Xie, Jiping Su

**Affiliations:** 1 Department of Otorhinolaryngology, Yulin Maternal and Child Health Care Hospital, Yulin, Guangxi, China; 2 Department of Otolaryngology-Head and Neck Surgery, The First Affiliated Hospital of Guangxi Medical University, Nanning, Guangxi, China

**Keywords:** breastfeeding, complementary food introduction, early-life nutrition, formula feeding, palatine tonsil grading

## Abstract

**Background:**

Early-life nutritional factors play a crucial role in child health, but their association with palatine tonsil grading is not well understood. This study aimed to investigate the relationship between early-life nutrition and palatine tonsil grading in preschool children.

**Methods:**

A cross-sectional study of 2,786 children from 17 kindergartens in Yulin City was conducted. Palatine tonsil grading was assessed via physical examination, and early-life nutritional data were collected through parent questionnaires, with 816 valid matches to examination records. Associations between early-life nutrition and palatine tonsil grading were analyzed using age-adjusted ordered logistic regression, stratified by sex.

**Results:**

Among 2,786 preschool children aged 2–7 years, palatine tonsillar hypertrophy (grades III–IV) was observed in 5.28%. Females aged 2–4 years had a significantly lower risk than males (OR = 0.438, *p* = 0.028), with no sex difference in older children. Univariate analysis of 454 males revealed no significant associations between feeding patterns (0–6, 6–12, 12–24 months), durations of breastfeeding and formula feeding, timing of formula milk introduction, or timing of complementary food introduction feeding and palatine tonsil grading. In 362 females, exclusive formula feeding during 0–6 months was significantly associated with increased risk of higher palatine tonsil grading (OR = 2.625, *p* = 0.013), whereas formula introduction at 6–12 months was linked to a decreased risk (OR = 0.494, *p* = 0.047). Multivariable analysis suggests a possible increased risk for exclusive formula feeding during the first 6 months (OR = 2.409, *p* = 0.066), whereas formula introduction between 6 and 12 months showed no statistically significant (OR = 0.729, *p* = 0.494).

**Conclusion:**

Our results suggested a possible association between exclusive formula feeding during 0–6 months and a higher palatine tonsil grading in females, while larger studies are needed for confirmation. Other early nutritional exposures showed no significant effect, and no associations were observed in males.

## 1 Introduction

Breastfeeding serves as the most critical source of nutrition and immunological support in infancy, providing a rich composition of microbiota, immune components, and bioactive factors (1, 2). The World Health Organization (WHO) and United Nations International Children’s Emergency Fund (UNICEF) recommend exclusive breastfeeding for the first 6 months of life, followed by continued breastfeeding alongside complementary foods for up to 2 years or longer (3). Numerous studies have shown that breastfeeding protects against various otorhinolaryngological diseases. For example, breastfeeding beyond 6 months reduces the risk of allergic rhinitis (4), and exclusive breastfeeding for 3 or 6 months lowers the incidence of acute otitis media (OR = 0.18 and 0.25, respectively) (5). However, these protective effects are disease-specific and not universal; evidence linking breastfeeding to reduced risk of sleep-disordered breathing or atopic conditions such as asthma and eczema is inconsistent (6, 7). Moreover, prolonged breastfeeding may increase certain risks, including early childhood caries after 12 months (8) and food allergies with breastfeeding beyond 6 months (4). Taken together, these findings highlight a complex, nuanced relationship between breastfeeding duration and child health outcomes.

While breastfeeding has been linked to protection against some otorhinolaryngological diseases, its influence on palatine tonsillar hypertrophy has not been well elucidated. Palatine tonsillar hypertrophy is a common otorhinolaryngological condition that can cause upper airway obstruction, which leads to symptoms including snoring, sleep-disordered breathing, and hypoxemia, thereby impairing sleep quality, physical growth, and overall quality of life. As such, it represents an important public health concern. Although alterations of the tonsillar microenvironment in chronic palatine tonsillitis and tonsillar hypertrophy have been documented (9, 10), the factors influencing tonsillar growth and grading remain incompletely understood. Notably, differences in oral microbiota composition between breastfed and formula-fed infants have been demonstrated (11), suggesting that breastfeeding may influence tonsillar development not only through immune protection but also via shaping the early colonization and balance of oropharyngeal microbiota.

Meanwhile, numerous studies suggest that the influence of early nutrition on immune and microbial development is modulated by sex (12). For example, breastfeeding reduces the risk of neonatal respiratory tract infection in female but not male infants (13). Similarly, a randomized placebo-controlled trial among Tanzanian mothers living with HIV found that perinatal and postnatal vitamin supplementation was associated with a 32% reduction in female infant mortality, with no corresponding benefit observed in males (14). Vitamin A supplementation administered alongside measles vaccination to children aged 6–23 months exhibited sex-specific immunomodulatory effects, including decreased leukocyte subsets in males and increased leukocyte counts and ex vivo IFN-*γ* production in females (15).

Given that the palatine tonsils serve as a key mucosal immune site at the interface of microbial exposure, it is plausible that early-life nutritional factors such as breastfeeding, formula feeding, and the timing of complementary food introduction influence palatine tonsillar growth and grading by modulating the oropharyngeal microbiota and immune development. However, to date, no studies have investigated the association between early-life nutrition and palatine tonsil grading. Recognizing established sex differences in immunity, this study employs sex-stratified analysis to explore the relationship between early-life nutritional factors and palatine tonsil grading in preschool-aged children, providing novel insights into the role of early-life nutrition in shaping palatine tonsillar development and hypertrophy.

## 2 Materials and methods

### 2.1 Study design and participants

**Figure 1:**
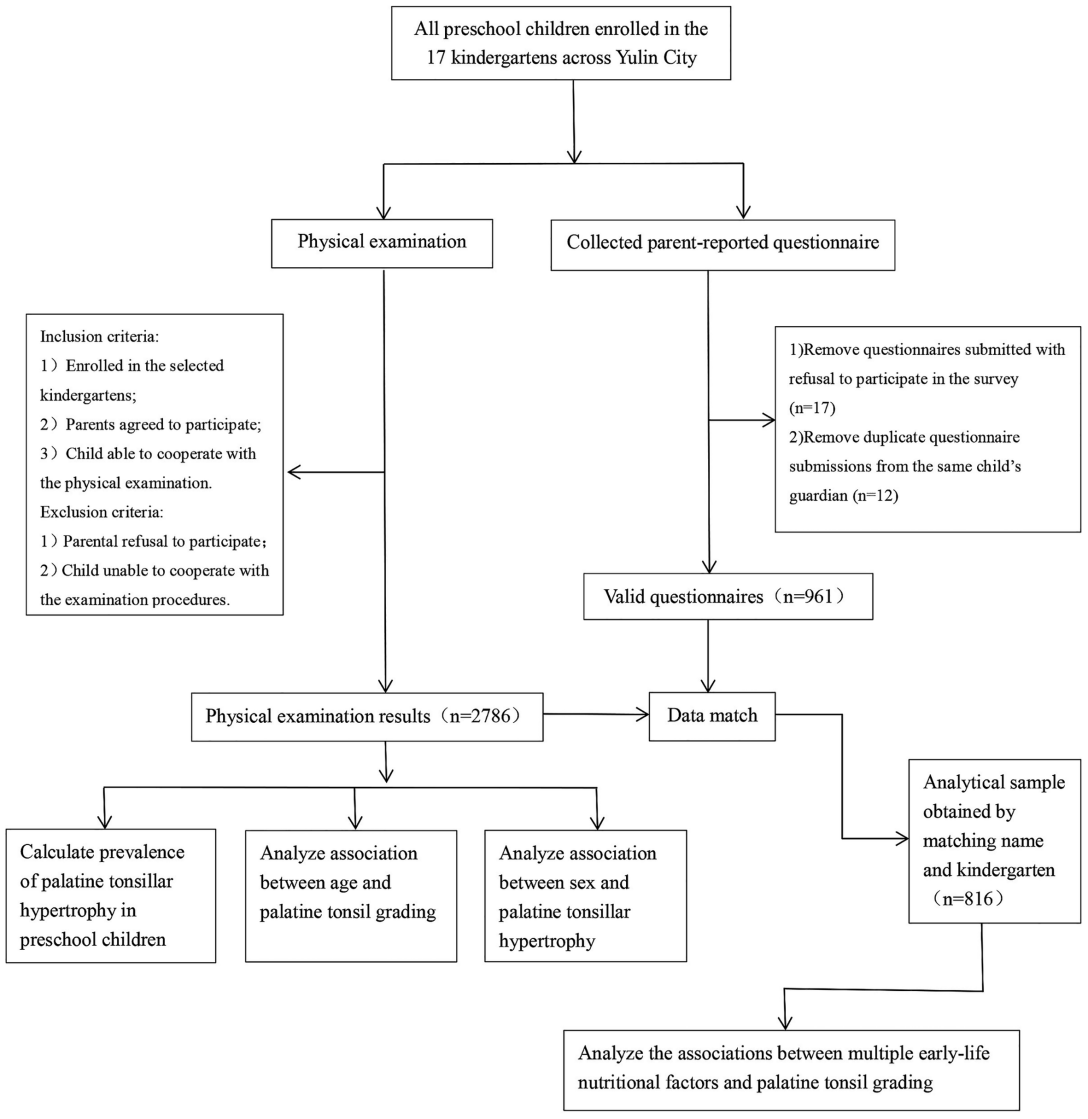
Flow diagram of the study.

Palatine tonsillar hypertrophy is common in preschool children, and insufficient parental awareness may lead to pediatric sleep-disordered breathing with consequent effects on growth and development. Our team coordinated with the local education department and kindergarten administrators to provide complimentary physical examinations for preschool children. The examination included assessment of the palatine tonsil grade, along with current height and weight. The study utilized a cluster sampling method to recruit participants from 17 kindergartens across the region. Before the examinations, kindergarten teachers informed parents about the screening initiative and obtained informed consent. Exclusion criteria: (1) parental refusal to participate in the physical examination; and (2) inability of the child to cooperate adequately with the examination procedures. Inclusion criteria: (1) enrolled in one of the 17 selected kindergartens; (2) parents agreed to participate; and (3) child able to cooperate with the physical examination. The specific research flowchart is presented in Figure 1. This study was reviewed and approved by the Medical Ethics Committee of the Yulin Maternal and Child Health Care Hospital, Guangxi, China (Approval No. [2025] Ethics Review (2)).

### 2.2 Data collection

Data were collected through structured physical examinations and parent-reported questionnaires from 2025-03-01 to 2025-05-30. The physical examinations were conducted by experienced otorhinolaryngologists. All grading was performed by visual inspection following a standardized procedure: with the child in an appropriate seated position, a tongue depressor was used to gently press down the anterior two-thirds of the tongue, allowing clear observation and assessment of the palatine tonsils. The size of the palatine tonsils was then recorded according to the Brodsky grading scale (16), which classifies tonsillar enlargement based on the percentage of oropharyngeal width occupied: Grade 0 (tonsils within the tonsillar fossa or previously removed), Grade I (<25%), Grade II (25–50%), Grade III (51–75%), and Grade IV (>75%). Tonsil size was expressed as the proportion of oropharyngeal space occupied. Palatine tonsillar hypertrophy was defined as >50% occupation of the oropharyngeal width, corresponding to Brodsky Grades III and IV.

Upon reaching each kindergarten, our team will coordinate with the teachers to simultaneously provide a parent-reported questionnaire, designed to collect data on early-life nutritional factors and other relevant information. Teachers will distribute the questionnaire link within their respective parent communication groups, enabling parents or guardians to complete it voluntarily and independently at their convenience. Before participation, all parents or guardians provided electronic informed consent through an online platform after receiving full information about the study. The questionnaire included basic demographic characteristics, perinatal-related characteristics, and detailed feeding history during infancy and early childhood. Key feeding variables were defined according to standardized criteria: breastfeeding duration was recorded as the age at the last breastfeeding episode; formula feeding variables included age at introduction and total duration, with formula intake defined as daily consumption of at least 300 mL; complementary foods were defined as all foods and beverages (excluding water, breast milk, and formula) introduced to supplement milk feeding. The timing of complementary food introduction was recorded as the age at which such foods or beverages were first regularly offered to the child. The full questionnaire is provided in Supplementary File 1.

### 2.3 Data security

To protect participant privacy, all collected questionnaire and physical examination data were assigned a unique study identification number after matching by name and kindergarten. Personally identifiable information was anonymized. All data were used solely for aggregate statistical analysis, and no individually identifiable information was disclosed in any public reporting of the study findings.

### 2.4 Variables and statistical analysis

In this cross-sectional study, descriptive statistics were first used to summarize palatine tonsil grading among the examined children, and the prevalence of palatine tonsillar hypertrophy was calculated. To evaluate the representativeness of the analytic sample, baseline characteristics of the questionnaire-included analytic sample were compared with those of the overall sample using chi-square tests for categorical variables (reported as frequencies and percentages). Palatine tonsil grading was treated as an ordered categorical variable (Grades 0–I, II, III, IV). The Spearman rank correlation coefficient was used to examine the association between palatine tonsil grading and age. Weight and height were regressed on age for each sex, and the standardized residuals from these models served as the age-independent Z-scores.

Feeding patterns were categorized according to age periods and feeding types. Specifically, feeding patterns during 0–6 months were classified as breastfeeding (exclusive breastfeeding from birth), formula feeding (exclusive formula feeding from birth), or mixed feeding (receipt of both breast milk and formula milk from birth). During both the 6–12 month and the 12–24 month periods, feeding patterns were classified as breastfeeding with complementary food (breast milk plus complementary foods, without formula), formula feeding with complementary food (formula plus complementary foods, without breast milk), or mixed feeding with complementary food (breast milk, formula, and complementary foods), respectively. Key early feeding variables were also included and categorized as follows: breastfeeding duration (<6 months, ≥6 to <12 months, ≥12 months), formula feeding duration (<12 months, ≥12 to <24 months, ≥24 months), timing of complementary food introduction (<5 months, ≥5 to <6 months, ≥6 to <7 months, ≥7 months), and timing of formula feeding initiation (no formula feeding, <6 months, ≥6 to <12 months, ≥12 months).

All models were adjusted for age to account for physiological age-related enlargement of tonsils. To explore potential sex-specific associations, analyses were stratified by sex, and separate ordinal logistic regression models were fitted for males and females. The proportional odds assumption was tested using the Brant test, and the omnibus test results are reported. Results are presented as odds ratios (ORs) with 95% confidence intervals (CIs). *p* value < 0.05 was considered statistically significant. All analyses and figure generation were performed using R software (version 4.4.1).

## 3 Results

### 3.1 Prevalence and trends related to age and sex in palatine tonsillar hypertrophy

**Figure 2:**
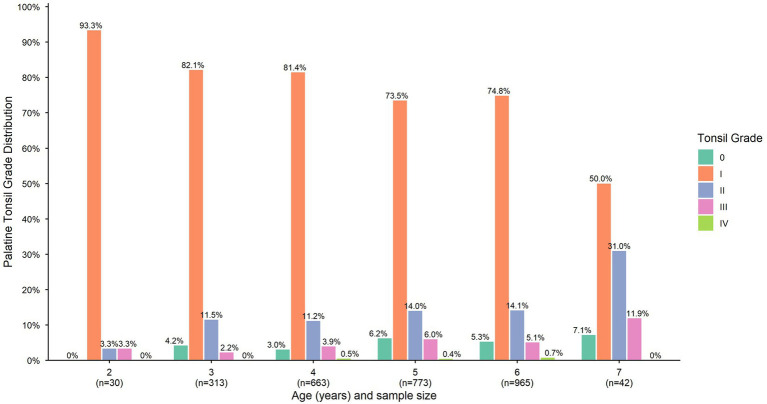
Distribution of palatine tonsil grading in preschool children (*n* = 2,786). Bars represent the percentage distribution of Brodsky tonsil grading within each age group, with respective sample sizes indicated below the age labels.

A total of 2,786 valid physical examination records were obtained from children enrolled in the 17 kindergartens. The distribution of palatine tonsil grading among the 2,786 preschool children was as follows: Grade 0 in 135 children (4.84%), Grade I in 2,136 (76.67%), Grade II in 368 (13.21%), Grade III in 134 (4.81%), and Grade IV in 13 (0.47%). The overall prevalence of palatine tonsillar hypertrophy (defined as Grade III or higher) was 5.28% (Figure 2). Age-stratified analysis revealed distinct trends in palatine tonsil grading, with the proportion of children exhibiting Grade 0 to I tonsils decreasing with age, while those with Grade II to IV tonsils increased. Spearman correlation analysis demonstrated a statistically significant positive association between tonsil grading and age (rho = 0.05, *p* < 0.01). Furthermore, ordinal logistic regression confirmed that increasing age was significantly associated with higher palatine tonsil grading, with an odds ratio of 1.123 (95% CI: 1.037–1.218, *p* < 0.01).

Subsequently, we conducted stratified analyses by age groups to examine the impact of sex on palatine tonsillar hypertrophy. In the 2 to 4-year-old group, females demonstrated a significantly lower risk compared to males (OR = 0.438, 95% CI: 0.21–0.915, *p* = 0.028), suggesting a protective effect of female sex in this age range. In contrast, no statistically significant difference was observed between females and males in the 5 to 7-year-old group (OR = 1.230, 95% CI: 0.836–1.809, *p* = 0.294). These results indicate that the association between sex and palatine tonsillar hypertrophy is age-dependent, with a significant protective effect of female sex apparent only in the younger age group.

### 3.2 Comparable baseline characteristics between analytic sample and overall cohorts

Online questionnaires were submitted by parents, yielding 990 responses. After excluding 17 submissions in which parents refused participation and 12 duplicate entries, 961 valid questionnaires were retained, corresponding to a valid response rate of 34.5% (961/2,786) relative to all children who underwent physical examination. Using kindergarten and child names as unique identifiers, these questionnaire data were matched to physical examination records, resulting in a final analytic sample of 816 children with complete data from both sources. To assess the representativeness of this analytic sample, baseline characteristics of these 816 preschool children were compared with those of the entire examination cohort (*n* = 2,786). No statistically significant differences were observed in sex, age, or tonsil-grade distribution between the two groups (*p* > 0.05) (Table 1), indicating that the analytic sample is representative of the overall study population and provides a valid foundation for subsequent association analyses.

**Table 1:** Baseline characteristics for the analytic sample and the entire cohort.

Characteristics	Overall cohort	Analytic sample	χ2	*P* value
	(*n* = 2786) No. (%)	(*n* = 816) No. (%)		
Sex			0.012	0.913
Male	1544 (55.4%)	454 (55.6%)		
Female	1242 (44.6%)	362 (44.4%)		
Age-tonsil grade (2–3 year)			NA	1.000*
0–II	335 (97.7%)	160 (97.6%)		
III–IV	8 (2.3%)	4 (2.4%)		
Age-tonsil grade (4–5 year)			0.087	0.769
0–II	1358 (94.6%)	356 (94.2%)		
III–IV	78 (5.4%)	22 (5.8%)		
Age-tonsil grade (6–7 year)			0.351	0.553
0–II	946 (93.9%)	260 (94.9%)		
III–IV	61 (6.1%)	14 (5.1%)		

* Fisher’s exact test. NA, not available.

### 3.3 Breastfeeding duration and adherence to WHO recommendations in the study population

**Figure 3:**
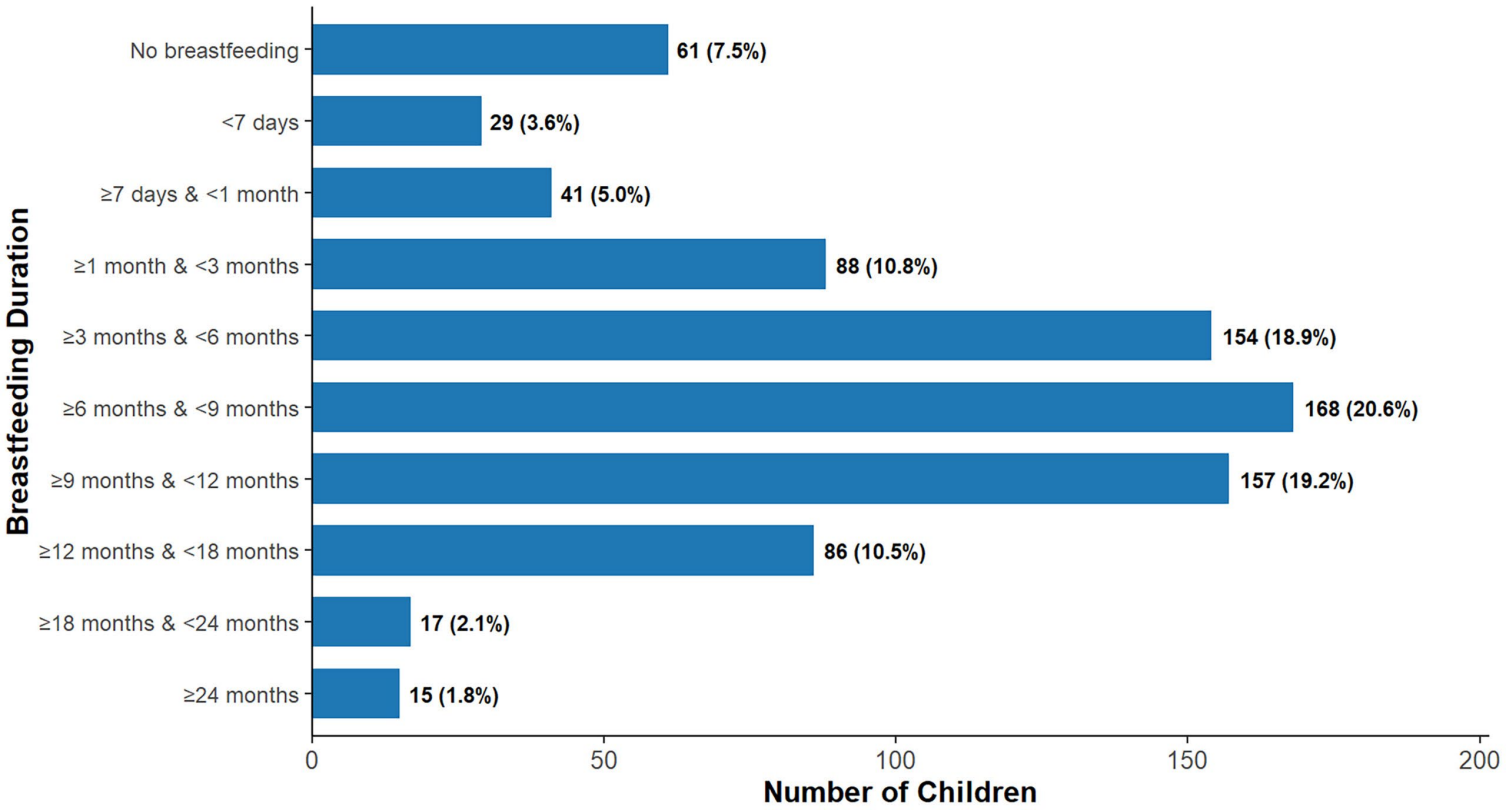
Distribution of breastfeeding duration among study participants (*n* = 816).

Analysis of breastfeeding practices among the 816 children revealed that a vast majority (92.5%) had been breastfed. However, compared with the WHO infant and young child feeding guidelines, which recommend exclusive breastfeeding for the first 6 months and continued breastfeeding up to 2 years or beyond, substantial gaps in adherence were evident (Figure 3). Specifically, 54.2% of children were breastfed for at least 6 months, while only 1.8% met the 24–month breastfeeding recommendation. Additionally, nearly one quarter (26.9%) of children were breastfed for less than 3 months. These findings suggest that although breastfeeding initiation rates are high in this population, maintaining breastfeeding for the recommended duration remains a significant challenge.

### 3.4 Associations between early feeding patterns and palatine tonsil grading

To investigate the association of feeding patterns before 2 years of age on palatine tonsil grading, including exclusive breastfeeding, formula feeding, mixed feeding, and introduction of complementary foods, we conducted age-adjusted ordered logistic regression analyses stratified by sex. Omnibus Brant tests for each feeding period and sex subgroup were nonsignificant (e.g., males 0–6 months: χ^2^ = 1.494, *p* = 0.684; females 0–6 months: χ^2^ = 2.251, *p* = 0.522), indicating no violation of the proportional odds assumption and supporting the use of ordered logistic regression. Among males, no feeding pattern during the studied age intervals (0–6 months, 6–12 months, and 12–24 months) showed a significant association with palatine tonsil grading. In females, exclusive formula feeding during the first 0–6 months was significantly associated with an increased risk of higher palatine tonsil grading (OR = 2.625, 95% CI: 1.228–5.61, *p* = 0.013), while other feeding patterns and age periods showed no significant relationships (Table 2).

**Table 2:** Sex-stratified associations of feeding patterns from 0 to 24 months with palatine tonsil grading.

Feeding pattern	Male (*N*=454)		Female (*N*=362)	
	OR (95%CI)	*p* value	OR (95%CI)	*p* value
0–6 months				
Breastfeeding	Reference		Reference	
Formula feeding	0.885 (0.385–2.035)	0.774	2.625 (1.228–5.61)	0.013
Mixed feeding	0.651 (0.345–1.231)	0.187	1.525 (0.834–2.789)	0.170
6–12 months				
Breastfeeding+Complementary food	Reference		Reference	
Formula feeding+Complementary food	0.806 (0.423–1.532)	0.510	1.616 (0.791–3.301)	0.188
Mixed feeding+Complementary food	0.518 (0.234–1.147)	0.105	1.242 (0.556–2.775)	0.598
12–24 months				
Breastfeeding+Complementary food	Reference		Reference	
Formula feeding+Complementary food	0.493 (0.176–1.383)	0.179	0.866 (0.236–3.183)	0.829
Mixed feeding+Complementary food	0.686 (0.208–2.261)	0.535	0.521 (0.107–2.535)	0.419

### 3.5 Associations of breastfeeding and formula feeding durations with palatine tonsil grading

To assess the association of breastfeeding and formula feeding durations on palatine tonsil grading, the Brant test for the proportional odds assumption indicated no violations in any subgroup. Specifically, for breastfeeding duration, the Omnibus Brant test showed χ^2^ = 1.025 (*p* = 0.795) in males and χ^2^ = 1.554 (*p* = 0.669) in females. For formula feeding duration, the corresponding Omnibus Brant statistics were χ^2^ = 1.264 (*p* = 0.737) in males and χ^2^ = 3.626 (*p* = 0.305) in females. Breastfeeding durations of 6–12 months and ≥12 months showed no significant association with palatine tonsil grading compared to <6 months in either sex. Similarly, formula feeding durations of 12–24 months and ≥24 months showed no significant association with palatine tonsil grading compared to <12 months in either sex (Table 3).

**Table 3:** Sex-stratified associations of early feeding durations with palatine tonsil grading.

Duration	Male (*N* = 454)		Female (*N* = 362)	
	OR (95%CI)	*p* value	OR (95%CI)	*p* value
Breastfeeding				
<6 months	Reference		Reference	
≥6 months & <12 months	1.132 (0.606–2.113)	0.697	0.713 (0.396–1.283)	0.259
≥12 months	1.513 (0.697–3.283)	0.295	0.82 (0.361–1.863)	0.636
Formula feeding				
<12 months	Reference		Reference	
≥12 months & <24 months	0.916 (0.386–2.172)	0.842	0.943 (0.427–2.083)	0.884
≥24 months	1.333 (0.701–2.536)	0.381	1.265 (0.679–2.356)	0.459

### 3.6 Association of timing of formula feeding and complementary food introduction with palatine tonsil grading

To further evaluate the associations between the timing of introducing formula milk or complementary foods and palatine tonsil grading. The proportional odds assumption was satisfied in all models, supported by Omnibus Brant tests showing non-significant results: formula milk introduction in males (χ^2^ = 2.395, *p* = 0.663) and females (χ^2^ = 2.724, *p* = 0.605), as well as complementary food introduction in males (χ^2^ = 4.013, *p* = 0.404) and females (χ^2^ = 0.761, *p* = 0.944). Notably, in females, introducing formula milk between 6 and 12 months was associated with a significantly lower risk of higher palatine tonsil grading (OR = 0.494, 95% CI: 0.246–0.989, *p* = 0.047) compared to introduction before 6 months. No statistically significant associations were observed between the timing of formula milk introduction and palatine tonsil grading in males. Additionally, the timing of complementary food introduction did not show any significant relationship with palatine tonsil grading in either males or females across all examined time categories (Table 4).

**Table 4:** Sex-stratified associations of feeding introduction timing with palatine tonsil grading.

Timing of introducing	Male (*N* = 454)		Female (*N* = 362)	
	OR (95%CI)	*p* value	OR (95%CI)	*p* value
Formula milk				
<6 months	Reference		Reference	
≥6 months & <12 months	1.166 (0.589–2.308)	0.659	0.494 (0.246–0.989)	0.047
≥12 months	2.207 (0.997–4.885)	0.051	0.843 (0.361–1.968)	0.693
No formula feeding	2.46 (0.762–7.941)	0.132	1.225 (0.372–4.032)	0.738
Complementary food				
<5 months	Reference		Reference	
≥5 months & <6 months	0.688 (0.298–1.59)	0.382	0.676 (0.275–1.661)	0.393
≥6 months & <7 months	0.494 (0.225–1.088)	0.080	0.944 (0.42–2.121)	0.889
≥7 months	0.69 (0.29–1.643)	0.401	1.295 (0.518–3.236)	0.581

### 3.7 Associations of demographic and perinatal factors with palatine tonsil grading

To explore whether demographic and perinatal factors are associated with palatine tonsil grading, model adequacy was confirmed using omnibus Brant tests for each variable within sex-stratified models. All tests yielded non-significant results (e.g., birth order in males: χ^2^ = 0.712, *p* = 0.700; in females: χ^2^ = 1.082, *p* = 0.582), supporting the use of ordinal logistic regression. Overall, most perinatal and demographic variables showed no statistically significant associations with tonsil grading (all *p* > 0.05). In the female subgroup, two marginal findings were observed: gestational age <37 weeks was associated with higher tonsil grading (OR = 2.735, 95% CI: 0.991–7.552, *p* = 0.052), while birth order ≥2 was associated with lower tonsil grading (OR = 0.584, 95% CI: 0.340–1.002, *p* = 0.051). Maternal history of upper respiratory infection during pregnancy, maternal age at pregnancy, mode of delivery, location of residence, birth weight, and age-adjusted weight and height Z-scores were not significantly associated with tonsil grading (Table 5).

**Table 5:** Sex-stratified associations of perinatal and demographic factors with palatine tonsil grading.

Characteristics	Male (*N* = 454)		Female (*N* = 362)	
	OR (95%CI)	*p* value	OR (95%CI)	*p* value
Maternal age at pregnancy (year)				
<25 y	Reference		Reference	
25–29 y	1.373 (0.643–2.93)	0.413	1.897 (0.818–4.402)	0.136
30–34 y	0.95 (0.421–2.144)	0.901	2.107 (0.88–5.046)	0.094
≥35 y	0.641 (0.193–2.122)	0.466	1.578 (0.501–4.963)	0.436
Maternal history of URI during pregnancy				
No	Reference		Reference	
Yes (≥1 episode)	0.943 (0.494–1.799)	0.859	0.822 (0.460–1.467)	0.507
Gestational age (week)				
≥37w & <42w	Reference		Reference	
<37w	1.013 (0.292–3.512)	0.984	2.735 (0.991–7.552)	0.052
≥42w	1.770 (0.486–6.441)	0.387	0.995 (0.207–4.775)	0.995
Birth order				
1^st^	Reference		Reference	
≥2^nd^	0.983 (0.557–1.735)	0.952	0.584 (0.340–1.002)	0.051
Birth weight				
≥2500g & <4000g	Reference		Reference	
<2500 g	1.614 (0.635–4.107)	0.315	0.686 (0.275–1.709)	0.418
≥4000 g	0.542 (0.125–2.353)	0.414	1.000 (0.271–3.686)	1.000
Mode of delivery				
Vaginal delivery	Reference		Reference	
Cesarean delivery	0.595 (0.289–1.222)	0.157	1.394 (0.773–2.514)	0.269
Location of residence				
Rural	Reference		Reference	
Urban	1.015 (0.577–1.785)	0.959	0.938 (0.546–1.614)	0.818
Weight Z-score (Per 1-SD increase)	0.940 (0.705–1.253)	0.672	0.939 (0.717–1.230)	0.649
Height Z-score (Per 1-SD increase)	1.049 (0.790–1.392)	0.742	0.976 (0.750–1.271)	0.857

URI, upper respiratory infection.

### 3.8 Independent early feeding factors and associations with palatine tonsil grading

A multivariable analysis was performed in females to assess the combined effects of feeding patterns during the first 0–6 months and the timing of formula milk introduction on palatine tonsil grading. The proportional odds assumption was satisfied (Omnibus Brant test χ^2^ = 4.57, *p* = 0.600). None of the formula milk introduction timing categories showed a statistically significant association with palatine tonsil grading. Compared to exclusive breastfeeding during 0–6 months, formula feeding showed a possible risk for increasing palatine tonsil grading, although this did not reach statistical significance (OR = 2.409, 95% CI: 0.943–6.152, *p* = 0.066). Mixed feeding was not significantly associated with palatine tonsil grading (OR = 1.454, 95% CI: 0.638–3.315, *p* = 0.373) (Table 6). However, given the low prevalence of palatine tonsillar hypertrophy (Grade III-IV, 5.28%) and the size of the subsample of females (*n* = 362), the study is likely to have low statistical power to detect associations, especially in multivariable analyses. The lack of observed associations (null statistical significance) could be due in part to insufficient power, not necessarily to the absence of a real association. The elevated point estimate associated with exclusive formula feeding during the first 6 months in females suggests a possible adverse effect that warrants further investigation.

**Table 6:** Early feeding patterns and formula introduction timing with palatine tonsil grading in females (*N* = 362).

Variables	OR (95%CI)	*p* value
Timing of introducing formula milk		
<6 months	Reference	
≥6 months & <12 months	0.729 (0.295–1.802)	0.494
≥12 months	1.124 (0.425–2.972)	0.813
No formula feeding	1.796 (0.459–7.027)	0.400
Feeding pattern (0–6months)		
Breastfeeding	Reference	
Formula feeding	2.409 (0.943–6.152)	0.066
Mixed feeding	1.454 (0.638–3.315)	0.373

## 4 Discussion

To our knowledge, this is the first study to illustrate the association between early life nutrition and palatine tonsil grading in preschool children, which provides new evidence for re-evaluating the role of early nutrition in tonsillar development. Our study showed no significant association between breastfeeding duration and palatine tonsil grading in children. This result contrasts with the well-established protective role of breastfeeding against other childhood otolaryngological disorders, such as otitis media and allergic rhinitis (4, 5). For example, Ding reported that breastfeeding beyond 6 months was associated with a reduced risk of allergic rhinitis (OR = 0.88, 95% CI: 0.79–0.98) (4). Furat K demonstrated that both exclusive breastfeeding for 3 months (OR = 0.18) and for 6 months (OR = 0.25) were significant protective factors against acute otitis media (5).

Previous studies have consistently demonstrated the protective effects of breastfeeding against infectious and allergic diseases (17–20). Bioactive components in breast milk, including interferon-gamma and long-chain polyunsaturated fatty acids, promote antigen-specific immune tolerance and modulate the Th1/Th2 balance by enhancing Th1 responses, thereby counteracting Th2-polarized allergic inflammation (19, 21). Furthermore, breastfeeding supports the establishment of a healthy infant gut microbiota. Microbial metabolites such as short-chain fatty acids can enhance the frequency and functional activity of regulatory T cells through epigenetic and other signaling pathways (22, 23). These mechanisms collectively contribute to the immunological profile observed in breastfed infants, characterized by higher proportions of regulatory T cells and lower levels of pro-inflammatory cytokines compared to formula-fed infants (24). In contrast, its impact on non-infectious, non-allergic health outcomes appears limited. For example, a large randomized controlled trial by Kramer found no significant developmental or behavioral benefits associated with prolonged breastfeeding (25), and extended breastfeeding did not effectively mitigate cardio metabolic risk in healthy term-born children (26). Furthermore, some cohort studies have even suggested potential adverse effects of prolonged breastfeeding duration: breastfeeding beyond 12 months may increase the risk of early childhood caries (27) and is associated with a higher risk of food allergy (RR = 2.41) (28).

However, the pathophysiology of tonsillar hypertrophy is fundamentally distinct from that of allergic diseases. The core pathological process in tonsillar hypertrophy appears to be hyperplasia of the lymphoid tissue itself, which is strongly influenced by genetic factors. Zupin showed that specific rare haplotypes of the DEFB1 gene—which encodes the antimicrobial peptide human beta-defensin-1 (hBD-1)—are significantly associated with susceptibility to tonsillar hypertrophy (OR = 9.90) (29). Additionally, Huang demonstrated that hypertrophic tonsil tissue exhibits extensive upregulation of innate immune and inflammatory pathways compared with normal tonsils, including enhanced activity in viral-sensing pathways (RIG-I/MAVS), multiple Toll-like receptors (TLR4/7), and key inflammatory mediators such as IL-1β, NF-κB, and IL-7 (30). When disease progression is driven predominantly by intrinsic genetic susceptibility or localized immune-microenvironment dysregulation, the systemic immunomodulatory effects conferred by breastfeeding are likely insufficient to counteract these strong inherent proliferative and inflammatory tendencies. Therefore, breastfeeding appears to exert only a limited influence on the proliferation of tonsillar lymphoid tissue.

We observed no relationship between palatine tonsil grading and formula feeding duration in preschool children, which is consistent with a non-significant association with breastfeeding duration. This parallel null result further supports the concept that palatine tonsillar hypertrophy is largely driven by genetic predisposition and intrinsic lymphoid hyperplasia. However, our findings reveal an interesting observation regarding the timing of formula feeding and its association with tonsillar hypertrophy risk, especially in females. Specifically, exclusive formula feeding during the first 6 months showed a significant association with increased risk of higher palatine tonsil grading in univariate analysis (OR = 2.625, *p* = 0.013), and maintained a similar but non-significant association after multivariable adjustment (OR = 2.409, *p* = 0.066) when compared to exclusive breastfeeding. These findings suggest that nutritional exposures during this critical early developmental period may influence tonsillar immune maturation and hypertrophy risk. This concept aligns with evolving pediatric allergy prevention strategies, where early introduction of allergens such as peanuts at 4–6 months has been shown to promote immune tolerance (31). Additionally, the observed sex-specific differences are consistent with previous reports in developmental nutrition. Nepal found that antenatal multiple micronutrient supplementation increased mean birthweight substantially in females but not males (32), and Friis observed a comparable female-predominant effect on birth length (33). These studies suggested that females may benefit more than males from maternal micronutrient supplements.

This sex-specific difference in biological response may be explained not only by nutritional factors but also by early-life immune development. For instance, Klein showed that males and females differ in their immunological responses to foreign and self-antigens (12). Bellamy reported significantly higher monocyte and basophil counts in male than female infants at 13 months of age (34). Lee demonstrated that natural killer cell frequencies are generally higher in male than female children (35). Furthermore, male infants have greater pro-inflammatory responses than females following stimulation with either lipopolysaccharide or mitogens (36). These observations collectively suggest that males develop a more robust innate immune profile than females in early life. Based on these findings, we propose that exclusive formula feeding during the first 6 months may increase the risk of higher tonsil grading in females owing to a double vulnerability: greater sensitivity to early nutritional factors and less robust innate immune protection during infancy. Nonetheless, given the borderline statistical significance in adjusted models, these results should be interpreted with caution. Larger, well-powered studies are needed to verify these observations and to further elucidate the underlying biological mechanisms.

Our study indicated that the timing of complementary food introduction did not significantly influence palatine tonsil grading in preschool children. This lack of significant association likely reflects the complexity of immune programming in response to diverse early antigen exposures. Complementary food introduction involves exposure to a broad spectrum of plant and animal proteins, along with various other food-derived components. The immunogenicity, diversity, and variability of these exposures are substantially greater than those associated with formula feeding alone. Such a strong multi-antigenic stimulus may dominate the tonsillar immune response, potentially overshadowing any timing-related effects. This interpretation is consistent with findings from several large cohort studies reporting no clear association between the timing of complementary feeding and immune-related outcomes such as allergic diseases (7, 37). In summary, these results suggest that in the context of highly diverse dietary antigen exposure, the window of introduction may be less critical for tonsillar immune maturation compared to more controlled, single-antigen exposures like formula feeding.

In this study, we found that the prevalence of palatine tonsillar hypertrophy in preschool children (2–7 years old) is 5.28%, with palatine tonsil grading showing an increasing trend across this age range. These findings are consistent with those reported by Akcay (38), who reported a palatine tonsillar hypertrophy prevalence of 3.4% in children aged 4–17 years and noted that tonsil size peaks between 4 and 8 years of age. As secondary lymphoid organs, tonsils primarily mediate B-lymphocyte proliferation in response to antigenic or polyclonal stimuli (39). During the preschool period, children’s expanding environmental exposures heighten antigenic challenge, which likely drives physiological hyperplasia of the tonsils as part of Waldeyer’s ring, providing a plausible immunological explanation for the observed peak in palatine tonsil grading during these years. It is worth noting that tonsillar development follows a distinct age-dependent trajectory, with physiological involution typically initiating around 10 years of age. This process is characterized by structural remodeling of the reticulated epithelium and reduced density of Langerhans cells, which together diminish antigen recognition and presentation capacity, ultimately lowering B-cell proliferation (39). Such involution may partially account for the somewhat higher prevalence detected in our study (5.28%) compared with Akcay (3.4%), as our cohort was limited to children aged 2–7 years, which is a period of active tonsil immune activity, and Akcay’s cohort included older children who have already entered the degenerative stage.

This study systematically examined the associations between early-life nutrition and palatine tonsil grading through a cross-sectional survey among preschool children. However, several limitations warrant consideration. According to anatomical location, tonsils can be divided into four types: palatine tonsils, pharyngeal tonsils (adenoids), lingual tonsils, and eustachian tube tonsils. In this study, we only focus on the palatine tonsils due to several methodological and clinical reasons. Palatine tonsils are the predominant site of acute and chronic tonsillitis and symptomatic hypertrophy in preschool children. Their superficial location allows standardized, large-scale clinical examination and objective size grading. In addition, indications and techniques for palatine tonsillectomy are well established, forming a relatively independent clinical framework. By contrast, assessment of other lymphoid tissues, such as adenoids, typically requires nasal endoscopy or X-radiographic evaluation, which is difficult for large-scale population-level physical examinations of preschool children.

The other limitation of this study is the retrospective collection of exposure data. All infant-feeding information was obtained via parental questionnaires, which introduces potential recall bias. Existing studies indicate that the accuracy of maternal recall for breastfeeding duration is influenced by sociodemographic factors, notably maternal education, household income, and urban versus rural residence. Haaga showed that women with little or no education and rural residents gave less reliable data on breastfeeding duration (40). Huttly found that others who were wealthier and more educated tended to overestimate the duration of breastfeeding during the follow-up (41). Li reported that the mothers tended to recall too early a date of introduction for formula milk (42). Furthermore, past exposures may be more vivid or meaningful to the case group than controls group (43). Parents of cases may recall past exposures more thoroughly and accurately, possibly due to heightened concern about their child’s condition or repeated questioning by health professionals. In contrast, parents of controls lack such prompts, tend to have vaguer memories, and may be more prone to systematic misreporting. Therefore, if control parents disproportionately misclassified formula introduction actually occurring after 6 months as ≤6 months (i.e., artificially inflating the apparent exposure prevalence in the control group), while case parents recalled more accurately and were thus less misclassified, the true exposure difference between cases and controls would be diluted, thereby underestimating the true strength of the association between exposure and outcome. This bias is particularly relevant to the findings in the female subgroup. Our study observed that females aged 2–4 years had a lower baseline risk of tonsillar hypertrophy than males (OR = 0.438), indicating that female cases already represent a group with lower underlying risk. Under this premise, the aforementioned recall bias operates similarly in the female subgroup (44); the observed association between exclusive formula feeding within the first 6 months and increased tonsillar grading among females (OR = 2.409, *p* = 0.066) is likely an underestimate of the true effect. The actual effect size may be larger than this point estimate and might reach conventional statistical significance if the bias were corrected. Despite these limitations, parental retrospective recall remains the most commonly used and practical method for assessing in pediatric epidemiological studies (45). We attempted to improve recall accuracy by using structured questionnaires with specific memory prompts. Nevertheless, full elimination of recall bias would require prospective data collection.

Additionally, all participants were recruited from a single geographic region, which may limit the generalizability of our findings to populations with differing cultural backgrounds or genetic profiles. Specifically, key factors such as infant feeding practices, population genetics, and the local prevalence of respiratory pathogens are likely to influence the result. Breastfeeding practices vary widely globally, with the highest prevalence of continued breastfeeding at 12 months observed in sub-Saharan Africa, South Asia, and parts of Latin America. In contrast, most high-income countries report rates below 20%, with notable disparities even among economically similar nations (46). Concurrently, the global epidemiology of respiratory pathogens shows substantial disparities in both incidence rate and timing of infection. More than 95% of respiratory syncytial virus (RSV)-associated acute lower respiratory infection episodes and more than 97% of RSV attributable deaths are concentrated in low- and middle-income countries (47). In addition, peak RSV incidence occurs earlier (0–3 months) in lower-income countries, whereas it occurs later (3–6 months) in higher-income settings (47). Last but not least, individuals from different populations vary considerably in their susceptibility to immune-related disease (48). Many associated genome-wide association (GWAS) variants show highly divergent allele frequencies (F_st_ > 0.4) across groups, supporting a history of population-specific selection (49). Thus, validation in more diverse cohorts is needed to confirm the wider applicability of these results.

Household socioeconomic status is a potential unmeasured confounder in this study. According to the World Bank classification for the 2024 fiscal year, China is currently classified as an upper-middle-income country (50), where formula feeding tends to be introduced earlier and more frequently in higher socioeconomic status families (51, 52). At the same time, higher socioeconomic status is generally associated with lower burdens of upper respiratory viral infections (47) and lower rates of household smoking exposure (53), both of which are established risk factors for tonsillitis (54, 55). Recurrent upper respiratory infections can lead to persistent antigenic stimulation and local immune dysregulation in the tonsils (55), while environmental tobacco smoke exposure has been linked to increased incidence of tonsillitis, snoring, adenoid hypertrophy, and a twofold higher risk of tonsillectomy (56–60). Thus, in our study population, if formula feeding is more prevalent among higher socioeconomic status families, the formula-fed group would contain a larger proportion of children with lower infection-related risk and lower smoking exposure. Both factors would independently reduce the apparent risk in the formula-fed group, thereby biasing the association between early formula feeding and tonsillar hypertrophy toward the null.

Although individual-level socioeconomic status indicators were not directly collected in this study, urban–rural residence was included as a geographic proxy variable. Univariate analysis showed no significant association between residential area and tonsillar grade, suggesting that, within the geographic scope of this study, socioeconomic status differences proxied by residential location may have a limited impact on effect estimates. In addition, urban–rural residence may also, to some extent, reflect potential differences in exposure to pesticides or environmental pollutants in rural industrial areas. The lack of a significant association between residential area and tonsillar grade in this study indirectly suggests that such environmental exposures, as captured by the urban–rural gradient, are unlikely to be major confounders of the observed associations. It should be noted that urban–rural classification is a crude proxy that neither fully substitutes for individual-level socioeconomic status indicators such as education, income, or occupation, nor precisely quantifies individual-level environmental exposure burden. Therefore, the possibility of residual confounding cannot be completely ruled out.

Finally, family history of tonsillar hypertrophy remains a potential unmeasured confounder, which was not collected in this study. Previous studies have demonstrated that breastfeeding for at least 6 months and the introduction of complementary solids between 4 and 6 months of age are effective strategies for allergy prevention (61). If a family history of palatine tonsillar hypertrophy is more prevalent in the case group and is similarly associated with longer breastfeeding duration or later formula introduction, the apparent exposure rate of “early formula feeding” in the case group would be systematically underestimated, thereby biasing the true association between exclusive formula feeding during the first 6 months and tonsillar grading toward the null. It should be noted that palatine tonsillar hypertrophy and allergic diseases are not completely equivalent in their pathogenesis, and direct evidence regarding the influence of family history of tonsillar hypertrophy on breastfeeding duration or timing of formula introduction is currently lacking. Therefore, the direction and magnitude of this unmeasured confounding on the effect estimates could not be quantitatively assessed.

## 5 Conclusion

Physiological age-related enlargement contributed to variations in palatine tonsil size during early childhood. Additionally, sex was associated with palatine tonsil grading, with females aged 2 to 4 years showing a lower risk of tonsillar hypertrophy compared to males. Most early-life feeding factors, including breastfeeding duration, formula feeding duration, timing of formula milk introduction, and timing of complementary food introduction, were not significantly associated with palatine tonsil grading in preschool children. However, exclusive formula feeding during the first 6 months showed a possible risk of increasing palatine tonsil grading in females, but this finding should be considered exploratory and hypothesis-generating, and requires validation in prospective cohorts with larger sample sizes and more rigorous exposure assessment.

## Data Availability

The raw data supporting the conclusions of this article will be made available by the authors, without undue reservation.

## References

[1] Hinde K German JB. Food in an evolutionary context: insights from mother's milk. J Sci Food Agric. (2012) 92:2219–23. doi: 10.1002/jsfa.5720, 22729619 PMC3836823

[2] Walker A. Breast milk as the gold standard for protective nutrients. J Pediatr. (2010) 156:S3–7. doi: 10.1016/j.jpeds.2009.11.021, 20105662

[3] World Health Organization (2018). Guideline: Counselling of Women to Improve Breastfeeding Practices. Geneva: World Health Organization 1. Available online at: https://www.ncbi.nlm.nih.gov/pubmed/30933442 (Accessed March 12, 2026). 30933442

[4] Ding Y Zhu C Li S Liu N Liu Q Li W . Breastfeeding and risk of food allergy and allergic rhinitis in offspring: a systematic review and meta-analysis of cohort studies. Eur J Pediatr. (2024) 183:3433–43. doi: 10.1007/s00431-024-05580-w, 38771371 PMC11263247

[5] Al-Nawaiseh FK Al-Jaghbir MT Al-Assaf MS Al-Nawaiseh HK Alzoubi MM. Breastfeeding initiation and duration and acute otitis media among children less than two years of age in Jordan: results from a case-control study. BMC Pediatr. (2022) 22:370. doi: 10.1186/s12887-022-03427-7, 35764971 PMC9238244

[6] Ponce-Garcia C Hernandez IA Major P Flores-Mir C. Association between breast feeding and paediatric sleep disordered breathing: a systematic review. Paediatr Perinat Epidemiol. (2017) 31:348–62. doi: 10.1111/ppe.12372 28590549

[7] Nwaru BI Craig LC Allan K Prabhu N Turner SW McNeill G . Breastfeeding and introduction of complementary foods during infancy in relation to the risk of asthma and atopic diseases up to 10 years. Clin Exp Allergy. (2013) 43:1263–73. doi: 10.1111/cea.12180, 24152159

[8] Lustosa K Rodrigues LRS Rocha RM Prudente TP Mezaiko E Silva FPY . Risk of early childhood dental caries associated with prolonged breastfeeding: a systematic review and Meta-analysis. Int J Paediatr Dent. (2025) 35:964–85. doi: 10.1111/ipd.13313, 40254914 PMC12332104

[9] Wu S Hammarstedt-Nordenvall L Jangard M Cheng L Radu SA Angelidou P . Tonsillar microbiota: a cross-sectional study of patients with chronic tonsillitis or tonsillar hypertrophy. mSystems. (2021) 6:e01302-20. doi: 10.1128/mSystems.01302-20, 33688019 PMC8547005

[10] Park J Lee KE Choi DH Kim YK Lee WH Kim MS . The association of tonsillar microbiota with biochemical indices based on obesity and tonsillar hypertrophy in children. Sci Rep. (2023) 13:22716. doi: 10.1038/s41598-023-49871-y, 38123635 PMC10733282

[11] Holgerson PL Vestman NR Claesson R Ohman C Domellof M Tanner AC . Oral microbial profile discriminates breast-fed from formula-fed infants. J Pediatr Gastroenterol Nutr. (2013) 56:127–36. doi: 10.1097/MPG.0b013e31826f2bc6, 22955450 PMC3548038

[12] Klein SL Flanagan KL. Sex differences in immune responses. Nat Rev Immunol. (2016) 16:626–38. doi: 10.1038/nri.2016.90, 27546235

[13] Sinha A Madden J Ross-Degnan D Soumerai S Platt R. Reduced risk of neonatal respiratory infections among breastfed girls but not boys. Pediatrics. (2003) 112:e303. doi: 10.1542/peds.112.4.e303, 14523216

[14] Kawai K Msamanga G Manji K Villamor E Bosch RJ Hertzmark E . Sex differences in the effects of maternal vitamin supplements on mortality and morbidity among children born to HIV-infected women in Tanzania. Br J Nutr. (2010) 103:1784–91. doi: 10.1017/S0007114509993862, 20211040 PMC3099235

[15] Jensen KJ Fisker AB Andersen A Sartono E Yazdanbakhsh M Aaby P . The effects of vitamin a supplementation with measles vaccine on leucocyte counts and in vitro cytokine production. Br J Nutr. (2016) 115:619–28. doi: 10.1017/S0007114515004869, 26678511

[16] Brodsky L. Modern assessment of tonsils and adenoids. Pediatr Clin N Am. (1989) 36:1551–69. doi: 10.1016/s0031-3955(16)36806-7, 2685730

[17] Bener A. Ehlayel M. S. Alsowaidi S. Sabbah A. (2007) Role of breast feeding in primary prevention of asthma and allergic diseases in a traditional society. Eur Ann Allergy Clin Immunol 39: 337–343. Available online at: https://www.ncbi.nlm.nih.gov/pubmed/18386435 (Accessed March 12, 2026). 18386435

[18] Fisk CM Crozier SR Inskip HM Godfrey KM Cooper C Roberts GC . Breastfeeding and reported morbidity during infancy: findings from the Southampton women's survey. Matern Child Nutr. (2011) 7:61–70. doi: 10.1111/j.1740-8709.2010.00241.x, 21143586 PMC6860776

[19] Hoang MP Samuthpongtorn J Seresirikachorn K Snidvongs K. Prolonged breastfeeding and protective effects against the development of allergic rhinitis: a systematic review and meta-analysis. Rhinology. (2022) 60:82–91. doi: 10.4193/Rhin21.274, 34783797

[20] PatnodeCD HenriksonNB WebberEM BlasiPR SengerCA Guirguis-BlakeJM. Breastfeeding and health outcomes for infants and children: a systematic review. Pediatrics. (2025) 156:e2025071516. doi: 10.1542/peds.2025-071516, 40240318

[21] Vieira Borba V Sharif K Shoenfeld Y. Breastfeeding and autoimmunity: Programing health from the beginning. Am J Reprod Immunol. (2018) 79:e12778. doi: 10.1111/aji.12778, 29083070

[22] Rooks MG Garrett WS. Gut microbiota, metabolites and host immunity. Nat Rev Immunol. (2016) 16:341–52. doi: 10.1038/nri.2016.42, 27231050 PMC5541232

[23] Chiu CY Liao SL Su KW Tsai MH Hua MC Lai SH . Exclusive or partial breastfeeding for 6 months is associated with reduced Milk sensitization and risk of eczema in early childhood: the PATCH birth cohort study. Medicine (Baltimore). (2016) 95:e3391. doi: 10.1097/MD.0000000000003391, 27082611 PMC4839855

[24] Wood H Acharjee A Pearce H Quraishi MN Powell R Rossiter A . Breastfeeding promotes early neonatal regulatory T-cell expansion and immune tolerance of non-inherited maternal antigens. Allergy. (2021) 76:2447–60. doi: 10.1111/all.14736, 33432577

[25] Kramer MS Fombonne E Igumnov S Vanilovich I Matush L Mironova E . Effects of prolonged and exclusive breastfeeding on child behavior and maternal adjustment: evidence from a large, randomized trial. Pediatrics. (2008) 121:e435–40. doi: 10.1542/peds.2007-1248, 18310164

[26] Martin RM Patel R Kramer MS Vilchuck K Bogdanovich N Sergeichick N . Effects of promoting longer-term and exclusive breastfeeding on cardiometabolic risk factors at age 11.5 years: a cluster-randomized, controlled trial. Circulation. (2014) 129:321–9. doi: 10.1161/CIRCULATIONAHA.113.005160, 24300437 PMC3946966

[27] Shrestha SK Arora A Manohar N Ekanayake K Foster J. Association of Breastfeeding and Early Childhood Caries: a systematic review and Meta-analysis. Nutrients. (2024) 16:1355. doi: 10.3390/nu16091355, 38732602 PMC11085424

[28] Matsumoto N Yorifuji T Nakamura K Ikeda M Tsukahara H Doi H. Breastfeeding and risk of food allergy: a nationwide birth cohort in Japan. Allergol Int. (2020) 69:91–7. doi: 10.1016/j.alit.2019.08.007, 31540813

[29] Zupin L Celsi F Bresciani M Orzan E Grasso DL Crovella S. Human beta defensin-1 is involved in the susceptibility to adeno-tonsillar hypertrophy. Int J Pediatr Otorhinolaryngol. (2018) 107:135–9. doi: 10.1016/j.ijporl.2018.01.041, 29501294

[30] Huang Q Hua H Li W Chen X Cheng L. Simple hypertrophic tonsils have more active innate immune and inflammatory responses than hypertrophic tonsils with recurrent inflammation in children. J Otolaryngol Head Neck Surg. (2020) 49:35. doi: 10.1186/s40463-020-00428-3, 32487224 PMC7268328

[31] Greer FR Sicherer SH Burks AW Committee on Nutrition; Section on Allergy and Immunology. The effects of early nutritional interventions on the development of atopic disease in infants and children: the role of maternal dietary restriction, breastfeeding, hydrolyzed formulas, and timing of introduction of allergenic complementary foods. Pediatrics. (2019) 143:e20190281. doi: 10.1542/peds.2019-0281 30886111

[32] Osrin D Vaidya A Shrestha Y Baniya RB Manandhar DS Adhikari RK . Effects of antenatal multiple micronutrient supplementation on birthweight and gestational duration in Nepal: double-blind, randomised controlled trial. Lancet. (2005) 365:955–62. doi: 10.1016/S0140-6736(05)71084-9, 15766997

[33] Friis H Gomo E Nyazema N Ndhlovu P Krarup H Kaestel P . Effect of multimicronutrient supplementation on gestational length and birth size: a randomized, placebo-controlled, double-blind effectiveness trial in Zimbabwe. Am J Clin Nutr. (2004) 80:178–84. doi: 10.1093/ajcn/80.1.178, 15213046

[34] Bellamy GJ Hinchliffe RF Crawshaw KC Finn A Bell F. Total and differential leucocyte counts in infants at 2, 5 and 13 months of age. Clin Lab Haematol. (2000) 22:81–7. doi: 10.1046/j.1365-2257.2000.00288.x 10792397

[35] Lee BW Yap HK Chew FT Quah TC Prabhakaran K Chan GS . Age- and sex-related changes in lymphocyte subpopulations of healthy Asian subjects: from birth to adulthood. Cytometry. (1996) 26:8–15. doi: 10.1002/(SICI)1097-0320(19960315)26:1<8::AID-CYTO2>3.0.CO;2-E, 8809475

[36] Casimir GJ Heldenbergh F Hanssens L Mulier S Heinrichs C Lefevre N . Gender differences and inflammation: an in vitro model of blood cells stimulation in prepubescent children. J Inflamm (Lond). (2010) 7:28. doi: 10.1186/1476-9255-7-28, 20525175 PMC2890631

[37] Obbagy JE English LK Wong YP Butte NF Dewey KG Fleischer DM . Complementary feeding and food allergy, atopic dermatitis/eczema, asthma, and allergic rhinitis: a systematic review. Am J Clin Nutr. (2019) 109:890S–934S. doi: 10.1093/ajcn/nqy220, 30982864

[38] Akcay A Kara CO Dagdeviren E Zencir M. Variation in tonsil size in 4- to 17-year-old schoolchildren. J Otolaryngol. (2006) 35:270–4. doi: 10.2310/7070.2005.0118, 17176803

[39] Siegel G. Theoretical and clinical aspects of the tonsillar function. Int J Pediatr Otorhinolaryngol. (1983) 6:61–75. doi: 10.1016/s0165-5876(83)80104-9, 6607904

[40] Haaga JG. Reliability of retrospective survey data on infant feeding. Demography. (1988) 25:307–14. doi: 10.2307/2061296 3396753

[41] Huttly SR Barros FC Victora CG Beria JU Vaughan JP. Do mothers overestimate breast feeding duration? An example of recall bias from a study in southern Brazil. Am J Epidemiol. (1990) 132:572–5. doi: 10.1093/oxfordjournals.aje.a115693, 2389760

[42] Li R Scanlon KS Serdula MK. The validity and reliability of maternal recall of breastfeeding practice. Nutr Rev. (2005) 63:103–10. doi: 10.1111/j.1753-4887.2005.tb00128.x 15869124

[43] Walter SD. Recall bias in epidemiologic studies. J Clin Epidemiol. (1990) 43:1431–2. doi: 10.1016/0895-4356(90)90113-4 2254782

[44] Cummings P Rivara FP Thompson RS Reid RJ. Ability of parents to recall the injuries of their young children. Inj Prev. (2005) 11:43–7. doi: 10.1136/ip.2004.006833, 15691989 PMC1730187

[45] Pless CE Pless IB. How well they remember. The accuracy of parent reports. Arch Pediatr Adolesc Med. (1995) 149:553–8. doi: 10.1001/archpedi.1995.02170180083016, 7735412

[46] Victora CG Bahl R Barros AJ Franca GV Horton S Krasevec J . Breastfeeding in the 21st century: epidemiology, mechanisms, and lifelong effect. Lancet. (2016) 387:475–90. doi: 10.1016/S0140-6736(15)01024-7 26869575, 26869575

[47] Li Y Wang X Blau DM Caballero MT Feikin DR Gill CJ . Global, regional, and national disease burden estimates of acute lower respiratory infections due to respiratory syncytial virus in children younger than 5 years in 2019: a systematic analysis. Lancet. (2022) 399:2047–64. doi: 10.1016/S0140-6736(22)00478-0, 35598608 PMC7613574

[48] Nedelec Y Sanz J Baharian G Szpiech ZA Pacis A Dumaine A . Genetic ancestry and natural selection drive population differences in immune responses to pathogens. Cell. (2016) 167:657–669 e621. doi: 10.1016/j.cell.2016.09.025, 27768889

[49] Brinkworth JF Barreiro LB. The contribution of natural selection to present-day susceptibility to chronic inflammatory and autoimmune disease. Curr Opin Immunol. (2014) 31:66–78. doi: 10.1016/j.coi.2014.09.008, 25458997 PMC4344185

[50] The World Bank (2026) World Bank Country and Lending Groups. Available online at: https://datahelpdesk.worldbank.org/knowledgebase/articles/906519-world-bank-country-and-lending-groups (Accessed March 12, 2026).

[51] Neves PA Barros AJ Baker P Piwoz E Santos TM Gatica-Dominguez G . Consumption of breast milk, formula and other non-human milk by children aged under 2 years: analysis of eighty-six low- and middle-income countries. Public Health Nutr. (2022) 25:680–8. doi: 10.1017/S1368980020004061, 33059789 PMC9991621

[52] Sarki M Parlesak A Robertson A. Comparison of national cross-sectional breast-feeding surveys by maternal education in Europe (2006-2016). Public Health Nutr. (2019) 22:848–61. doi: 10.1017/S1368980018002999, 30516455 PMC6474715

[53] Hiscock R Bauld L Amos A Fidler JA Munafo M. Socioeconomic status and smoking: a review. Ann N Y Acad Sci. (2012) 1248:107–23. doi: 10.1111/j.1749-6632.2011.06202.x, 22092035

[54] DiFranza JR Aligne CA Weitzman M. Prenatal and postnatal environmental tobacco smoke exposure and children's health. Pediatrics. (2004) 113:1007–15. doi: 10.1542/peds.113.s3.1007, 15060193

[55] Mitchell RB Archer SM Ishman SL Rosenfeld RM Coles S Finestone SA . Clinical practice guideline: tonsillectomy in children (update). Otolaryngol Head Neck Surg. (2019) 160:S1–S42. doi: 10.1177/0194599818801757, 30798778

[56] Willatt DJ. Children's sore throats related to parental smoking. Clin Otolaryngol Allied Sci. (1986) 11:317–21. doi: 10.1111/j.1365-2273.1986.tb00132.x, 3780018

[57] Said G Zalokar J Lellouch J Patois E. Parental smoking related to adenoidectomy and tonsillectomy in children. J Epidemiol Community Health (1978). (1978) 32:97–101. doi: 10.1136/jech.32.2.97, 681592 PMC1060925

[58] Hinton AE Herdman RC Martin-Hirsch D Saeed SR. Parental cigarette smoking and tonsillectomy in children. Clin Otolaryngol Allied Sci. (1993) 18:178–80. doi: 10.1111/j.1365-2273.1993.tb00824.x, 8365003

[59] Strachan DP Cook DG. Health effects of passive smoking. 4. Parental smoking, middle ear disease and adenotonsillectomy in children. Thorax. (1998) 53:50–6. doi: 10.1136/thx.53.1.50, 9577522 PMC1758689

[60] Stahlberg MR Ruuskanen O Virolainen E. Risk factors for recurrent otitis media. Pediatr Infect Dis J. (1986) 5:30–2. doi: 10.1097/00006454-198601000-00006, 3945573

[61] Heine RG. Preventing atopy and allergic disease. Nestle Nutr Inst Workshop Ser. (2014) 78:141–53. doi: 10.1159/000354954, 24504215

