# Supplementary material for: The association between early-life nutrition and palatine tonsil grading in preschool children: a cross-sectional study
Source: Front Nutr. 2026 Mar 19;13:1789543. doi: 10.3389/fnut.2026.1789543 (PMC13043436; doi:10.3389/fnut.2026.1789543)
Supplement: Supplementary file 1 [file Table_1.DOCX]

**Survey Questionnaire: The Influence of Early-life Nutrition and Perinatal Factors on Tonsil Grading in Children: A Cross-Sectional Study**

NO.

The Department of Otorhinolaryngology at Yulin Maternity and Child Health Hospital is conducting a research study on the relationship between early-life nutrition, perinatal factors, familial predisposition, and tonsillar hypertrophy in preschool children. We aim to investigate how early feeding practices (for example, duration of breastfeeding and formula feeding, timing of formula introduction, and timing of complementary foods), perinatal factors (including gestational age, mode of delivery, birth weight, etc.), and family history of tonsillar conditions relate to tonsillar enlargement and general health outcomes (such as frequency of fever and hospitalizations). The goal of this research is to provide scientific evidence to support pediatric health management. Participation involves completing a questionnaire and allowing us to use relevant health examination data to analyze factors that may influence tonsillar development. We warmly invite you to take part in this survey. This study has been approved by the Ethics Committee of Yulin Maternity and Child Health Hospital. All information collected will be handled in accordance with ethical review standards. Personal details will be anonymized, and all data will be kept strictly confidential and used only for medical research purposes.

**Are you aware of and agree to participate in this survey? (Please check):**

**O Agree O Disagree**

***Basic information of the child:：***

1. Gender:  **O** Male **O** Female

2. Date of birth (XXXX year XX month): year month （Gregorian calendar）

3. Ethnicity？ **O** Han **O** Zhuang **O** Other （ ethnic）

4. Household registration？ **O** Rural **O** Urban

5. What is the gestational age？

**O** Premature birth (gestational age<37 weeks)

**O** Full term delivery (gestational age of 37 weeks but less than 42 weeks)

**O** Expired delivery (gestational age ≥ 42 weeks)

6. Delivery method?  **O** Vaginal delivery **O** Cesarean section

7. What is the number of births at birth？  **O** First tire **O** Second tire  **O** Third tire and above

8. Was it a multiple pregnancy at birth？ **O** Single tire **O** Twin tire  **O** Multiple tire

9. Weight at birth? **O**＜2500g **O**≥2500g and＜4000g **O**≥4000g

***Feeding situation:***

10. What is the feeding method for children under 6 months old？

**O** Breastfeeding  **O** Formula feeding **O** Breastfeeding and formula feeding

11. What was the feeding method when the child was 6 months to 1 year old？

**O** Breastfeeding + complementary foods  **O** Formula feeding + complementary foods

**O** Mixed breastfeeding and formula feeding + complementary foods

***Complementary foods refer to foods or beverages other than water, breast milk, or formula.**

12. What was the feeding method when the child was 1 to 2 years old?

**O** Breastfeeding + complementary foods  **O** Formula feeding + complementary foods

**O** Mixed breastfeeding and formula feeding + complementary foods

13. Up to which month did the child receive breastfeeding?

**O** No breastfeeding  **O**＜7 days **O**≥7 days and <1 month

**O**≥1 month and <3 months **O**≥3 months and <6 months **O**≥6 months and＜9 months  **O**≥9 months and＜12 months **O**≥12 months and＜18 months

**O**≥18 months and＜24 months  **O**≥24 months

***Breastfeeding duration refers to the age at the child’s last breastfeeding.**

14. At which month after birth did the child start formula feeding?

**O** No formula feeding **O**＜7 days  **O** ≥7 days and＜1 month

**O** ≥1 month and＜3 months **O** ≥3 months and＜6 months  **O** ≥6 months and＜9 months

**O**≥9 months and＜12 months  **O**≥12 months and＜18 months **O**≥18 months and＜24months  **O**≥24 months

***The start time of formula feeding refers to the age at the child’s first formula feeding.**

15. Up to which month did the child receive formula feeding?

**O** No formula feeding  **O**<1 month  **O**≥1 month and <3 months

**O**≥3 months and <6 months **O**≥6 months and <9 months  **O**≥9 months and <12 months

**O**≥12 months and <18 months **O**≥18 months and <24 months  **O**≥24 months

***Formula-feeding duration is counted only when daily formula intake is ≥300mL. If formula intake is less than 300 mL/day, it is not counted toward formula-feeding duration.**

16. At which month after birth was complementary food first introduced?

**O** <4 months **O**≥4 months and <5 months  **O** ≥5 months and <6 months

**O**≥6 months and <7 months **O**≥7 months

***The time of complementary food introduction refers to the age when complementary foods were first given to the child.**

***Health-related information:***

17. Does the child have a history of recurrent tonsillitis? If yes, please select the corresponding frequency.

**O** No  **O** <3 episodes/year  **O** 3–4 episodes/year

**O** 5–6 episodes/year **O**≥7 episodes/year **O** Unsure

18. Has the child ever undergone tonsil or adenoid surgery?

**O** No  **O** Unilateral tonsillectomy **O** Bilateral tonsillectomy **O** Adenoidectomy

19. On average, how many febrile episodes (body temperature ≥38°C) has the child had per year since birth?

**O** 1–2 episodes/year **O** 3–4 episodes/year **O** 5–6 episodes/year  **O**≥7 episodes/year

20. How many times has the child been hospitalized in total since birth?

**O** Never hospitalized **O** 1–2 times  **O** 3–4 times  **O** 5–6 times **O**≥7 times

**Hospitalization — specific reason(s) (single choice, multiple choice, or write‑in allowed):**

- Acute pharyngitis
- Bronchitis
- Pneumonia
- Abdominal pain
- Diarrhea
- Adenoid hypertrophy
- Tonsillar hypertrophy
- Acute tonsillitis
- Chronic tonsillitis
- Meningitis
- Congenital heart disease
- Tracheitis
- Other

***Family information:***

1. ***Mother’s information***

21. Mother’s age during pregnancy?

**O** <25 years  **O**≥25 and <30 years  **O**≥30 and <35 years

**O**≥35 and <40 years  **O**≥40 years

22. Any upper respiratory tract infections during pregnancy? (e.g., nasal congestion, sore throat, fever, coughs)

**O** No **O** 1–2 times **O**≥3 times

23. History of recurrent tonsillitis? (≥3 episodes/year) **O** No  **O** Yes

24. Ever undergone tonsillectomy?  **O** No  **O** Yes

1. ***Father’s information***

25. Ever undergone tonsillectomy?  **O** No  **O** Yes

26. History of recurrent tonsillitis? (≥3 episodes/year)  **O** No **O** Yes

1. ***Siblings (If the child is an only child, you may skip the following questions.)***

27. History of recurrent tonsillitis? (≥3 episodes/year)

**O** No **O** Yes

28. Ever undergone tonsillectomy?

**O** No **O** Yes

**This concludes the questionnaire. Thank you again for your support!**
